# Nanomaterial-encapsulated STING agonists for immune modulation in cancer therapy

**DOI:** 10.1186/s40364-023-00551-z

**Published:** 2024-01-07

**Authors:** Xi Chen, Zhijie Xu, Tongfei Li, Abhimanyu Thakur, Yu Wen, Kui Zhang, Yuanhong Liu, Qiuju Liang, Wangrui Liu, Jiang-Jiang Qin, Yuanliang Yan

**Affiliations:** 1 Department of Pharmacy, Xiangya Hospital, Central South University, 410008 Changsha, Hunan China; 2 Department of Pathology, Xiangya Hospital, Central South University, 410008 Changsha, Hunan China; 3 National Clinical Research Center for Geriatric Disorders, Xiangya Hospital, Central South University, 410008 Changsha, Hunan China; 4 Hubei Key Laboratory of Embryonic Stem Cell Research, School of Basic Medical Sciences, Hubei University of Medicine, 442000 Shiyan, Hubei China; 5 Pritzker School of Molecular Engineering, Ben May Department for Cancer Research, University of Chicago, 60637 Chicago, IL USA; 6 National Engineering Research Center of Personalized Diagnostic and Therapeutic Technology, Furong Laboratory, Central South University, 410008 Changsha, Hunan China; 7 Department of Interventional Oncology, Renji Hospital, Shanghai Jiao Tong University School of Medicine, 200127 Shanghai, China; 8 Hangzhou Institute of Medicine (HIM), Zhejiang Cancer Hospital, Chinese Academy of Sciences, 310022 Hangzhou, Zhejiang China

**Keywords:** cGAS-STING pathways, STING agonists, Nanoparticles, Cancer immunotherapy

## Abstract

The cGAS-STING signaling pathway has emerged as a critical mediator of innate immune responses, playing a crucial role in improving antitumor immunity through immune effector responses. Targeting the cGAS-STING pathway holds promise for overcoming immunosuppressive tumor microenvironments (TME) and promoting effective tumor elimination. However, systemic administration of current STING agonists faces challenges related to low bioavailability and potential adverse effects, thus limiting their clinical applicability. Recently, nanotechnology-based strategies have been developed to modulate TMEs for robust immunotherapeutic responses. The encapsulation and delivery of STING agonists within nanoparticles (STING-NPs) present an attractive avenue for antitumor immunotherapy. This review explores a range of nanoparticles designed to encapsulate STING agonists, highlighting their benefits, including favorable biocompatibility, improved tumor penetration, and efficient intracellular delivery of STING agonists. The review also summarizes the immunomodulatory impacts of STING-NPs on the TME, including enhanced secretion of pro-inflammatory cytokines and chemokines, dendritic cell activation, cytotoxic T cell priming, macrophage re-education, and vasculature normalization. Furthermore, the review offers insights into co-delivered nanoplatforms involving STING agonists alongside antitumor agents such as chemotherapeutic compounds, immune checkpoint inhibitors, antigen peptides, and other immune adjuvants. These platforms demonstrate remarkable versatility in inducing immunogenic responses within the TME, ultimately amplifying the potential for antitumor immunotherapy.

## Introduction

Over the decades, immunotherapy has emerged as a promising option for several advanced and refractory tumors. Various immunotherapeutic approaches, such as immune checkpoint inhibitors (ICIs), vaccines, and chimeric antigen receptor (CAR) T-cell therapy, have been investigated in the preclinical and clinical stages for different malignancies [1]. However, the success of immunotherapy is still limited, mainly due to insufficient immune responses. A common obstacle to effective cancer immunotherapy is the presence of an immunosuppressive tumor microenvironment (TME). The TME is a complex entity consisting of immune cells, fibroblasts, endothelial cells, vasculature, cytokines, and chemokines. These components interact with tumor cells and are critical in tumor progression and therapeutic effects [2, 3]. For example, within the TME, immunostimulatory dendritic cells (DCs) can experience deficiencies or dysfunction, impacting T-cell proliferation and promoting heightened immune evasion across various cancer types [4]. Furthermore, specific inflammatory cytokines, such as interferon (IFN) and tumor necrosis factor-alpha (TNF-α), are primarily orchestrated by DCs and tumor-associated macrophages (TAMs) in the TME, and significantly influence tumor progression and occurrence [5]. In addition, TME components can inhibit drug penetration, leading to reduced responses to immunotherapy [6]. These challenges underscore the need to explore novel strategies for modulating TME to improve antitumor immunity.

In recent years, the cyclic GMP-AMP synthase (cGAS)-stimulator of interferon genes (STING) signaling pathway has emerged as a crucial player in cancer immunity. Activation of the STING signaling pathway can stimulate innate inflammatory immune responses and potentially overcome immunosuppression in TME [7]. In particular, STING activation can induce DC activity, leading to infiltration of IFN-γ-producing T cells in colorectal cancers [8]. Furthermore, STING-mediated type I interferon signaling amplifies the stem cell-like CD8^+^ T cell differentiation program, enhancing the generation of stem-like central memory CD8^+^ T cells from cancer tissues [9]. The prospects of STING agonism in antitumor immunotherapy have been steadily increasing. Several natural and synthetic STING agonists have been discovered, developed, and evaluated in preclinical models and clinical trials for multiple cancer therapies [10]. For instance, several cocktail strategies containing STING agonists (e.g. Mn^2+^ and MSA-2) and immune checkpoint inhibitors (e.g. YM101) have been demonstrated to conquer resistance to immunotherapy and generate broad-spectrum antitumor properties in preclinical studies [11, 12]. In a phase I clinical trial, Mn^2+^ (a potent cGAS-STING activator) plus anti-PD-1 antibody exhibited encouraging antitumor activities and favorable safety profiles in patients with advanced metastatic solid tumors [13]. However, conventional STING agonists often face challenges such as random diffusion, rapid clearance, and limited membrane permeability, leading to toxic cytokine storms and reduced bioavailability, compromising their clinical translation [14]. Therefore, there is a pressing need to explore new strategies to improve immunotherapeutic efficacy and reduce the adverse effects of STING agonists.

Nanotechnology has rapidly developed and are widely used in the biomedical field. Numerous nanoparticles (NPs) are used in cancer therapy to encapsulate and efficiently deliver active pharmaceutical ingredients to tumor sites [15, 16]. Various nanotechnology platforms, such as liposomes, polymersomes, and metal-based nanomedicines, have been approved by the US Food and Drug Administration (FDA) to load antitumor agents such as Doxil, DaunoXome, and Onivyde [17]. Recently, a new trend has emerged that focuses on nanoplatforms that encapsulate STING agonists using organic and inorganic nanomaterials. These therapeutic NP platforms are designed to promote tumor penetration and accumulation, enhance cellular uptake, and reduce the rapid degradation of STING agonists.

Regarding antitumor immunity, NPs that carry STING agonists have shown promising results. They effectively activate the STING signaling pathway, improving the innate and adaptive immune responses of various cancers [18]. This review highlights the potential prospects and advantages of STING-activating nanoparticles (STING-NPs). We discuss the robust immune modulation exerted by STING-NPs on the TME composition. Furthermore, we emphasize the pleiotropic antitumor immunity achieved by co-delivering nanoplatforms incorporating STING agonists and other antitumor agents. Based on current investigations, STING-NPs present a promising new approach to cancer immunotherapy, offering a novel perspective to explore more effective treatments.

## Overview of cGAS-STING pathways in mediating immune responses

The discovery of cGAS-STING pathways has accelerated significantly in the past decade, making them a promising and potent area of research in the field of the immune system. As a sensor for cytosol DNA, activation of STING signaling can strongly induce innate immune programming in various diseases characterized by the expressions of interferons and pro-inflammatory cytokines (Table 1). In addition, STING-inducible innate immune responses are crucial in activating antigen-presenting cells (APCs) and T-cell priming, promoting adaptive immunity in anticancer therapy. Recent advances in our understanding of the molecular biology of the cGAS-STING pathway have highlighted the potential for pharmacological regulation, making STING an attractive target for modulating the immune response. In particular, the development of small-molecule STING agonists has emerged as a promising strategy for cancer immunotherapy.

**Table 1:** The major components of STING pathway

Components	Characteristics	Role in STING pathway
cGAS	An evolutionarily conserved cytosolic DNA sensor	It uses ATP and GTP as substrates to catalyze the synthesis of cGAMP
cGAMP	A cyclic dinucleotide second messenger	It binds to STING localized on the ER membrane, which promotes STING oligomerization and translocation from the ER to the Golgi apparatus
STING	An ER membrane protein as a key adaptor in innate immunity,	Its oligomerization recruits TBK1 and further triggers IRF3- and NF-κB-dependent transcription of type I IFNs
TBK1	A serine/threonine kinase	It phosphorylates STING and subsequently activates both IRF3 and NF-κB
IRF3	A transcription regulator	After phosphorylated by TBK1, it subsequently translocates to the nucleus, where it induces the transcription of inflammatory factors
NF-κB	A transcription regulator	After activated byTBK1 and IκB kinase epsilon, it synergizes with IRF3 to induce the transcription of inflammatory factors
Type I IFNs	Major downstream molecules (IFN-α and IFN-β) of STING pathway	IFN-α and IFN-β involve in host innate immune activation
Other proinflammatory factors	Inflammatory cytokines (such as TNF, IL-6 and IL-12) and chemokines (such as CXCL9 and CXCL10)	The STING activation is also required for the induction of many other genes encodings proinflammatory factors that regulates both innate and adaptive immunity

*Abbreviation*: STING: stimulator of interferon genes; cGAS: cyclic guanosine monophosphate–adenosine monophosphate (GMP-AMP) synthase; ATP: adenosine triphosphate; GTP: guanosine triphosphate; cGAMP: cyclic GMP-AMP; TBK1: TANK-binding kinase 1; ER: endoplasmic reticulum; IRF3: interferon regulatory factor 3; NF-κB: nuclear factor kappa-B; Type I IFNs: type I Interferons

### Activation of cGAS-STING

**Fig. 1:**
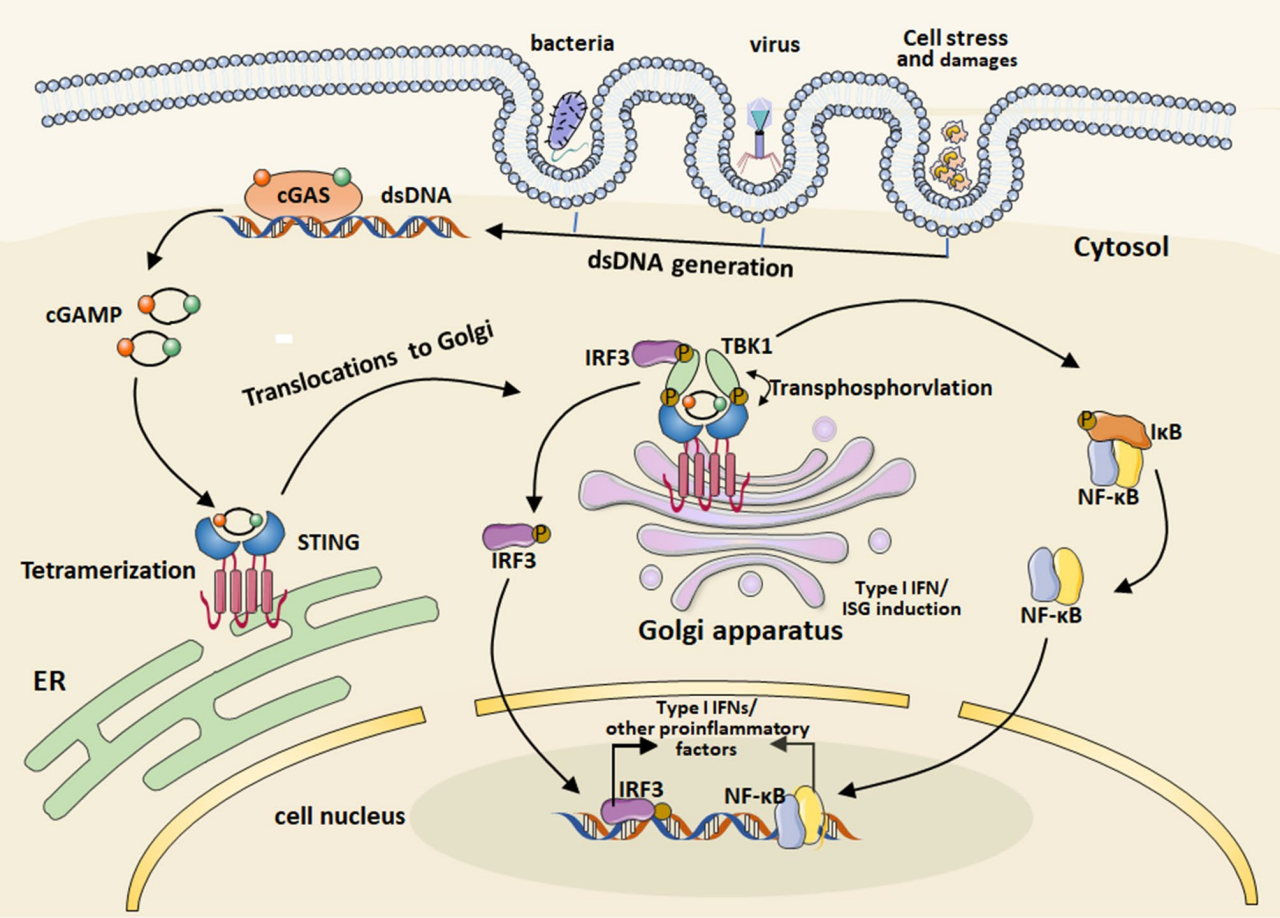
The activation of cGAS-STING pathways mediating the cytosolic nucleic acid sensing and innate immunity

The cGAS-STING pathway regulates both tumors and immune cells through innate immune mechanisms [19]. It can be activated by various factors, including pathogen detection, cellular stress, and damage, leading to the accumulation of cytosolic double-stranded DNA (dsDNA) [20]. Among these pathways, cGAS acts as a central cellular cytosolic dsDNA sensor. When binding to dsDNA, cGAS undergoes a conformational change and further catalyzes the production of second messenger cyclic GMP-AMP (cGAMP) [21]. Synthesized from ATP and GTP, cGAMP interacts with STING, an endoplasmic reticulum (ER) membrane protein. This interaction triggers a high-order oligomerization of STING, followed by its translocation from the ER to the Golgi apparatus and post-Golgi compartments [22]. In Golgi, STING oligomerization recruits TANK binding kinase 1 (TBK1), which transphosphorylates STING, leading to the recruitment of interferon regulatory factor 3 (IRF3) [23]. TBK1 further facilitates the phosphorylation of IRF3, causing its dimerization and translocation to the nucleus. This process stimulates the expression of type I IFNs and other pro-inflammatory genes [24]. Furthermore, TBK1 recruitment also phosphorylates IκB kinase, stimulating nuclear factor-κB (NF-κB). The translocation of NF-κB and IRF3 into the nucleus promotes the transcription of type I IFN and the induction of the interferon-stimulated genes [25, 26] (Fig. 1).

As the primary production of STING activation, type I IFNs mainly include IFN-α and IFN-β, which interact with heterodimer interferon receptors (IFNαR), activating both innate and adaptive immunity [27]. After activation of the cGAS-STING pathways, type I interferon production is instrumental in DC maturation and its cross-presentation of tumor antigens (TAs) to antigen-specific CD8^+^ T cells, which exerts adaptive antitumor immunity [28, 29]. Other innate immune cells, such as natural killer (NK) cells and neutrophils, are also increased in TME, with enhanced antitumor responses after activation of the STING pathways [30, 31]. Immunosuppressive cells also convert their characteristics by activating STING, such as reducing myeloid-derived suppressor cells and repolarizing macrophages [32, 33]. Therefore, STING-mediated production of IFNs reforms the immunogenicity of TME and increases the antitumor immune response.

### The development of STING agonists

Researchers have been devoted to exploring a wide range of STING agonists in recent years, continuously evaluating their potential in antitumor treatments (Table 2). Several small-molecule agents, such as cyclic dinucleotides (CDNs), are potential ligands directly targeting the STING protein. As the main constituents of STING agonists, CDNs can function as a second messenger to activate innate immune responses through IRF3-dependent production of type I IFNs [34]. Natural CDNs consist of exogenous small molecules generated from bacteria, such as cyclic dimeric guanosine monophosphate (c-di-GMP, CDG), cyclic dimeric adenosine monophosphate (c-di-AMP, CDA), 3′3′-cyclic AMP-GMP (3′3′-cGAMP), and endogenous 2′3′-cGAMP produced in mammalian cells [35]. These CDNs improve immunogenicity and tumor suppression in mouse cancer models, such as melanoma, breast, and colorectal cancers [36–38]. However, natural CDNs typically exhibit hydrophilic and electronegative properties and are susceptible to rapid enzymatic degradation, limiting their penetration and bioavailability in tumor treatment [39]. Some attempts are being made to address these challenges, such as developing synthetic CDNs and vehicles or particles to target delivery in tumor tissues [40].

**Table 2:** STING agonists involving in clinical development

Agents	molecular types	Route	With or without combinational agents	Targeting cancers	Phase	Clinical Trial NCT Code
ADU-S100 /MIW815	CDN analog	IT	alone or + Ipilimumab	Advanced/​metastatic solid tumors or lymphomas	Phase 1	NCT02675439
		IT	+PDR001	Advanced/​metastatic solid tumors or lymphomas	Phase 1/1b	NCT03172936
		IT	+Pembrolizumab	HNSCC	Phase 2	NCT03937141
BMS-986,301	CDN analog	IV/IT/ IM	Alone or+ Nivolumab and Ipilimumab	Advanced solid tumors	Phase 1	NCT03956680
BI-1,387,446	CDN analog	IT	Alone or + Ezabenlimab	Advanced/​metastatic solid tumors	Phase 1	NCT04147234
IMSA-101	CDN analog	IT	Alone or + ICI/+IO	Advanced solid tumors or lymphomas	Phase 1/2	NCT04020185
		IT	+PULSAR, Pembrolizumab and Nivolumab	NSCLC and RCC	Phase 2	NCT05846646
		IT	+PULSAR, Pembrolizumab and Nivolumab	Oligoprogressive solid tumors	Phase 2	NCT05846659
MK-1454	CDN analog	IT	+ Alone or Pembrolizumab	Advanced/​metastatic solid tumors or lymphomas	Phase 1	NCT03010176
		IT	+Pembrolizumab	Metastatic or unresectable, recurrent HNSCC	Phase 2	NCT04220866
SB11285	CDN analog	IV	Alone or + Atezolizumab	Advanced solid tumors	Phase 1	NCT04096638
TAK-676	CDN analog	IV	+ Pembrolizumab	NSCLC, TNBC and HNSCC	Phase 1	NCT04879849
		IV	Alone or + Pembrolizumab	Advanced/​metastatic solid tumors	Phase 1	NCT04420884
E7766	non-CDN molecule	IT	Alone	NMIBC	Phase 1/1b	NCT04109092
		IT	Alone	Advanced/​metastatic solid tumors or lymphomas	Phase 1/1b	NCT04144140
GSK3745417	non-CDN molecule	IV	Alone	AML and HR-MDS	Phase 1	NCT05424380
		IV	Alone or + Dostarlimab	Advanced solid tumors	Phase 1	NCT03843359
HG-381	non-CDN molecule	IV	Alone	Advanced solid tumors	Phase 1	NCT04998422
KL340399	non-CDN molecule	IT	Alone	Advanced solid tumors	Phase 1	NCT05549804
SNX281	non-CDN molecule	IV	Alone or + Pembrolizumab	Advanced solid tumors or lymphomas	Phase 1	NCT04609579
TAK-500	antibody drug conjugate	IV	Alone or + Pembrolizumab	Advanced/​metastatic solid tumors	Phase 1/2	NCT05070247
SYNB1891	Engineered bacteria vectors	IT	Alone or + Atezolizumab	Advanced/​metastatic solid tumors or lymphomas	Phase 1	NCT04167137

*Abbreviations*: CDN: cyclic dinucleotide; HNSCC: head and neck squamous cell carcinoma; NSCLC: non-small cell lung cancer; RCC: renal cell carcinoma; TNBC: triple-negative breast cancer (TNBC); NMIBC: non-muscle invasive bladder cancer; AML: acute myeloid leukemia; HR-MDS: High-risk myelodysplastic syndrome; ICI: immune checkpoint inhibitor; IO: Immuno-oncology; PULSAR: personalized ultra-fractionated stereotactic adaptive radiotherapy; IT: intratumoral; IV: intravenous; IM: intramuscular

In addition to CDNs, the pharmacological properties of non-CDN agents have become increasingly apparent in the activation of cGAS-STING. DMXAA, a classical model drug directly targeting murine STING, has shown disruption in the tumor vasculature [41]. Unfortunately, DMXAA did not succeed in clinical applications, as it does not bind to the human STING protein [42]. Based on the DMXAA structure, its analogs and derivatives have inspired the potential development of novel antitumor STING agonists [43, 44]. Furthermore, Ramanjulu et al. reported a novel non-CDN, diABZI, developed based on symmetry-related amidobenzimidazole (ABZI) [45]. This novel agonist demonstrates a high binding affinity to human STING and has shown promising results by inducing systemic tumor regression in mice with colorectal cancer [45]. Soon afterward, more stable non-CDN STING agonists have been discovered, such as SR-717 and MSA-2, which have high affinities for STING and show favorable antitumor potencies [46, 47]. Although these approaches have achieved satisfactory progress, the limitations of these small-molecule STING agonists, such as random dissemination, rapid clearance, and low accumulation at tumor sites, restrict the efficacy of existing immunotherapies [48]. There has been a growing interest in utilizing NPs for packaging small-molecule STING agonists. These NPs offer superior cellular uptake and improved tumor accumulation. As a result, nanotechnology holds the potential to overcome the limitations of free STING agonists. It offers a promising approach to enhance their effectiveness, a concept that will be examined more extensively below.

## Nanocarriers for encapsulating STING agonists

**Fig. 2:**
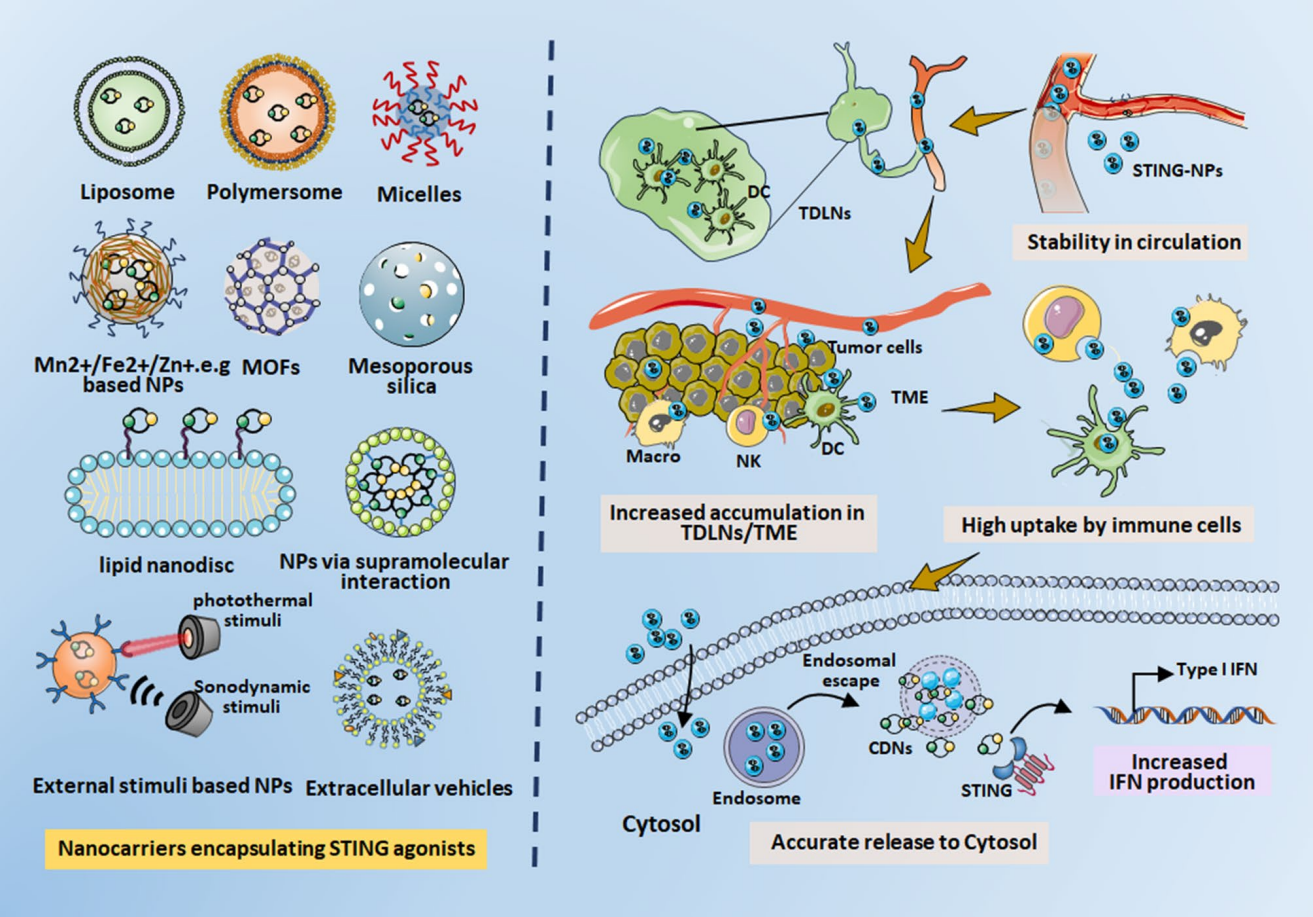
The overview presenting each type of nanocarriers encapsulating the STING agonists, and highlighting the advantages of STING-NP administration in aspects of stability, tumor/LNs accumulation, cellular uptake and release

Despite the profound antitumor immune responses produced by current small-molecule STING agonists, their clinical translation faces challenges, including rapid clearance, low efficacy for tumor targeting, and the risk of random diffusion leading to unwanted autoimmune toxicity. In response to these challenges, scientists have recently directed their attention toward creating diverse nanomaterials featuring unique surface modifications. These modifications aid in encapsulating and precisely delivering STING agonists to particular locations, such as TMEs and LNs. NPs that transport STING agonists offer several advantages over their free counterparts, including improved biocompatibility and greater flexibility in intracellular delivery. These attributes contribute to heightened STING-mediated immune responses (Fig. 2). In the following sections, we will briefly introduce the compositions, properties, and advantages of nanocarriers with incorporated STING agonists, along with their effects on STING-related agonism. Furthermore, we outline the current nanomaterials for encapsulation of STING agonists in Table 3.

**Table 3:** The nanomaterials for delivery of STING agonists

Nanomaterials	Encapsulated STING agonists	Encapsulated anti-tumor agents	Key design features	Targeting components of STING pathways	in vivo model	Ref
Liposome	cGAMP	N/A	Mannose-coated NPs for DCs’ reorganization and uptake	Upregulation of IFN-β and IL-12	V600E BRAF-mutated and B16F10 melanoma model	[53]
	cGAMP	N/A	Phosphatidylserine-coated surface for APCs’ reorganization and uptake	Upregulation of IFN-β, TNF-α, IL-6, p-TBK1 and p-IRF3	4T1 breast cancer and B16-OVA melanoma model	[54]
	cGAMP	N/A	A photothermal segment (FeS_2_) for generating hydroxyl radic; bromelain-modified surface for ECM degradation	Upregulation of TNF-α and IL-6	4T1 breast cancer model	[56]
	CDG	MPLA	The hydrophilic CDG into the NP core and hydrophobic MPLA into the lipid bilayer	Upregulation of IFN-β and TNF-α	B16F10 melanoma, 4T1 and D2.A1 breast cancer model	[138]
	CDG	MPLA	The hydrophilic CDG into the NP core and hydrophobic MPLA into the lipid bilayer	Upregulation of IFN-β	Panc02 pancreatic cancer model	[137]
	diABZI	αPD-L1 and αCD47	A lipid NP with dual linkages of αCD47 and αPD-L1	Upregulation of TNF-α, CXCL9 and CXCL10	CT-2 A and PVPF8 glioma model	[129]
Polymersomes	cGAMP	N/A	pH-sensitive segments (DEAEMA) with hydrophobic BMA for endosomal escapes	Upregulation of IFN-β, CXCL9 and CXCL10	B16F10 melanoma model	[59, 62]
	cGAMP	N/A	pH-sensitive segments (DEAEMA) with hydrophobic BMA for endosomal escapes	Upregulation of IFN-β, TNF-α, IL-12, CXCL10 and p-IRF3	B16F10 melanoma model	[61]
	cGAMP	N/A	pH-sensitive segments (DEAEMA) with hydrophobic BMA for endosomal escapes	Upregulation of IFN-β, CXCL10, TNF, IL-12, p-IRF3	Neuro-2a and 9464D neuroblastoma model	[60]
	cGAMP	Tumor antigen (OVA)	pH-sensitive segments (DEAEMA) with hydrophobic BMA for endosomal escapes	Upregulation of IFN-α, IFN-β, TNF-α and IL-6	B16F10 melanoma and MC38 colorectal cancer model	[133]
Micelles	cGAMP	N/A	PC7A NP as a polyvalent STING agonist for amplifying activation of STING pathway	Upregulation of IFN-β and CXCL10	MC38 colorectal cancer model	[68]
	cGAMP	Tumor antigen (OVA)	A cationic PDMA for electrostatic complexation with negatively charged cGAMP; a pH-responsive segment (PDPA) for NPs’ cellular disassembly and endosomal escapes	Upregulation of IFN-β, TNF-α, CXCL9, CXCL10 and IL-12	EG7 lymphoblastoma, MC38 colorectal and TC-1 cervical cancer model	[69]
Other Polymeric NPs	DMXAA	Tumor antigen (OVA)	Co-assembly of PEG-b-PDPA diblock copolymer with OEI-C14 for facilizing endosome escapes; surface modification with mannose for APCs’ reorganization	Upregulation of IFN-β, TNF-α, IL-6, CXCL10, Isg-15, p-IRF3 and p-TBK1	B16-OVA melanoma and 4T1 breast cancer model	[132]
	DMXAA	SN38	SN38-grafted block as a chemotherapeutic prodrug; amine-containing DEAEMA group providing a positive charge for effective encapsulation and cellular internalization	Upregulation of IFN-β, TNF-α, CXCL9, CXCL10 and IRF7	B16F10 melanoma and 4T1 breast cancer model	[63]
	CDNs	N/A	Cationic PBAEs as carriers for binding CDNs, specific cellular uptake and effective endosomal escapes	Upregulation of IRF3	B16F1 melanoma model	[86]
	diABZI	αPD-L1 and Gemcitabine	αPD-L1-coated surface for enhancing cellular uptake and antitumor efficacy; Gemcitabine-PLA segment as a chemotherapeutic prodrug	Upregulation of IRF7, IL-6, CXCL9, CXCL10 and IFN‐β	Panc02 pancreatic cancer, B16F10 melanoma and 4T1 breast cancer model	[125]
	cGAMP	siSIRPα	cationic lipid DOTAP and PEG-b-PLGA forming NPs that enhances encapsulation efficiency and cellular uptake	Upregulation of IFN-α and IFN-β	B16F10 melanoma model	[87]
	3′3 cGAMP	CpG ODNs, 5′ppp-dsRNA, and tumor antigen (TRP2)	carboxylic acid-terminated PLGA for preventing aggregation and embolisms upon intravenous injection	Upregulation of TNF-α and IL-12	B16F10 melanoma model	[88]
	CDG	αCD47	MPC as a zwitterionic polymer for superior BBB penetration; a FAP-α-responsive crosslinker targeted by the FAP-α enzyme in TME for effective release of CDG	Upregulation of IFN-β and TNF-α	GL261 glioma model	[127]
	cGAMP	Tumor antigen (OVA)	Self-degradable framework PBAEs for endosomal escape	Upregulation of IFN-β, TNF-α, CXCL9 and CXCL10	B16F10 melanoma model	[89]
Mn^2+^-based NPs	CDA	N/A	Mn^2+^ as a STING agonist that self-assembles with CDNs and amplifies STING activation	Upregulation of IFN-β, TNF-α, CXCL-9 and CXCL10	B16F10 melanoma and CT26 colorectal cancer model	[71]
INOP-based NPs	MSA-2	Tumor antigen (OVA)	Acid INOPs for augmenting STING activation and endosomal escapes	Upregulation of IFN-β, TNF-α, IL-6 and CXCL10	B16-OVA melanoma and MC38 colorectal cancer model	[72]
Zinc-based NPs	CDA	N/A	A non-toxic zinc phosphate hydrophilic core with surrounding lipid bilayer	upregulation of IFN-β, TNF-α and IL-6	B16F10 melanoma. MC38 colorectal cancer and GL261 glioma model	[73]
MOFs	SR-717	N/A	A photosensitizer (TCPP) for controlling oxidation-responsive SR-717 release	Upregulation of IFN-β and IL-6	4T1 breast cancer model	[78]
	DMXAA	CpG ODNs	MOF-801 as a STING agonist that self-assembles with DMXAA and CpG ODNs	Upregulation of IL-6, TNF-α, and cGAS-STING-NF-κB signaling	Hepa1-6 hepatoma carcinoma model	[141]
Mesoporous silica	CDG	N/A	Amine-modified surface electrostatically interacts with the anionic phospholipid membrane and ECM for enhancive local adherence	Upregulation of TNF-α	B16F10 melanoma model	[81]
	CDG	N/A	PEGylated modification and quaternary ammonium-modified surface for stable blood circulation, enhancive tumor accumulation, and cellular uptake	Upregulation of IL-1β, IFN-β, IL-6 and p-STING	4T1 breast cancer model	[82]
	CDG	N/A	Amine functionalization facilitating high CDG loading and effective release of CDG upon cellular internalization.	Upregulation of IFN-β	GL261 glioma model	[83]
	CDA	N/A	A larger pore size (5–10 nm) and a thinner Si-O-Si matrix for rapid release of CDA	Upregulation of IFN-β, CXCL10, CCL2, and CCL5	B16F10 melanoma model	[84]
Sono-driven NPs	MSA-2	N/A	A semiconducting polymer as a sonosensitizer that links with MSA-2 through a singlet oxygen cleavable linker (diphenoxyethene)	Upregulation of IFN-β, p-TBK1 and p-IRF3	SCC-7 head and neck squamous cell carcinoma model	[91]
Lipid nanodisc (LND)	CDNs	N/A	A flexible high-aspect-ratio morphology for improving penetration capacity; the conjugation of CDN prodrug and LND could be cleaved by cathepsin after cellular internalization	Upregulation of IFN-β, TNF-α and IL-6	MC38 colorectal cancer, TC-1 cervical cancer and 4T1 breast cancer model	[93]
Supramolecular NP	CDG	N/A	A hydrophobic nucleotide lipid (3’,5’-diOA-dC) assembling with CDG through various supramolecular forces	Upregulation of IFN-β, TNF-α, CXCL9, CXCL10, STING and p-IRF3	B16F10 melanoma model	[95]
Extracellular vehicles (EVs)	CDNs	N/A	EVs as a delivery vehicle with favorable biocompatibility	upregulation of IFN-β, CXCL-9 and CXCL10	B16F10 melanoma and CT26 colorectal cancer model	[97]

*Abbreviations*: p-TBK1: phosphorylated TBK1; p-IRF3: phosphorylated IRF3; p-STING: phosphorylated STING; OVA: ovalbumin; ECM: extracellular matrix; αCD47: CD47 antibody: αPD-L1: PD-L1 antibody; DEAEMA: 2-diethylaminoethyl methacrylate; BMA: butyl methacrylate; PBAEs: Poly (beta-amino esters); PLA: poly(lactic acid); MPC: 2-methacryloyloxyethyl phosphorylcholine; FAP-α: fibroblast activation protein α; PDMA: poly((2-dimethylaminoethyl) methacrylate; PDPA: poly(2-(diisopropylamino)ethyl methacrylate); INOPs: Acid-ionizable iron NPs; MOFs: metal–organic framework; TCPP: meso-tetra(carboxyphenyl)porphyrin; PLGA: poly(lactic-co-glycolic acid); DOTAP: 1,2-dioleoyl-3-trimethylammonium-propane; PEG: polyethylene glycol; Isg-15: Interferon stimulated gene 15

### Liposome

Liposomes are spherical-shaped artificial vesicles with one or more phospholipid bilayers surrounding a central aqueous compartment [49, 50]. Based on their amphipathic characteristics, liposomes have become promising drug delivery systems in various therapeutic areas [51]. In 1995, the FDA approved the first liposome product (Doxil®) to treat patients with ovarian cancers, highlighting its wide clinical application for cancer therapy [52]. Recently, chemical optimization in liposomes has been explored to enhance tumor penetration, intracellular uptake, and cytosol release of STING agonists. For example, cGAMP was encapsulated in DOTAP/cholesterol liposomes, preventing cGAMP from enzymatic degradation and systemic clearance. These liposomes were coated with mannose on their surface, enabling specific delivery of cGAMP to DCs, through targeting the abundant expression of mannose receptors on DCs. Mannose-coated liposomes with cGAMP encapsulation improved uptake by DCs, thus inducing pro-inflammatory cytokines related to STING compared to free cGAMP [53]. In another example, Liu et al. developed a cGAMP-loaded liposome that was surface modified with phosphatidylserine (PS) to enhance its recognition by PS receptors located in APCs. Furthermore, they established an interaction between PS and calcium ions (Ca^2+^) to facilitate the precipitation of cGAMP within the liposomal core using calcium phosphate (CaP). Once engulfed by APCs, the liposomes were disassembled at acidic endosomal pH, during which cGAMP was accurately released into the cytosol, thus promoting the initialization of STING signaling and the production of type I IFNs [54]. In addition to pH-responsive manners, the photothermally responsive liposome could also improve drug accumulation in tumor tissues by converting external light into heat [55]. Zhan et al. constructed a photothermally responsive liposome coated with ferrous sulfide (FeS_2_) that absorbs near-infrared (NIR). The photothermally responsive liposome shell could be melted by NIR, causing an effective accumulation of cGAMP in tumor tissues with a potent activation of the STING pathways [56]. It is important to note that incomplete drug release may still occur in this liposome formulation, highlighting the need for further strategies to enhance drug release efficiency. Additionally, careful control of the temperature is crucial when using photothermally sensitive liposomes. Excess thermal doses run the risk of inducing necrosis in normal tissues surrounding tumors [56].

### Polymersome

Polymersomes are amphiphilic vesicles developed from the self-assembly of diblock or triblock copolymers [57]. Most reported polymersomes contain an aqueous core for efficient drug incorporation, with a morphology similar to that of liposomes [57, 58]. Furthermore, the assembly of copolymers can be tailored to align with specific external stimuli-responsive requirements, allowing customized properties. To load STING agonists, several studies have used an amphiphilic diblock copolymer (poly(ethylene glycol)-block-[(2-diethylaminoethyl methacrylate)-co-(butyl methacrylate)-co-(pyridyl disulfide ethyl methacrylate)]) (PEG-DBP) to form a polymersome [59–62]. The PEG-DBP vesicle membrane was designed for pH-responsive disassembly, which further orchestrated endosomal escape and cytosolic delivery of STING agonists. In vitro and in vivo assays revealed that treatment of cGAMP-loaded PEG-DBP could create highly efficient uptake by APCs and NK cells and a broad distribution in tumor-draining lymph nodes (TDLNs). In a murine melanoma model, cGAMP-loaded PEG-DBP elevated inflammatory STING-driven production, while unencapsulated cGAMP barely triggered a response above baseline [59]. Similar to polymersomes, other polymeric NPs that are made up of blocks have shown enormous potentials for delivery of STING agonists. For instance, Liang et al. constructed a two-in-one polymeric NPs from triblock copolymers (poly(ethylene glycol)-block-poly-(DTMASN38)-block-poly[2-(diethylamino)-ethyl methacrylate]) (PEG-PSN38-PDEA) for the co-delivery of DMXAA and the prodrug of the chemotherapeutic agent SN-38. PEG-PSN38-PDEA comprised a cleavable prodrug (PSN38) to trigger redox stimuli in tumors, but its building block also formed a hydrophobic inner core during self-assembly. This nanoencapsulation further increased the cellular content of DMXAA in APCs and caused immunostimulation of the type I IFN pathway in murine melanoma. This block copolymers represents a novel design concept for a co-delivery nanoplatform that could lead to synergistic antitumor responses [63]. However, it is essential to note that this NP formulation involves more than two components, which may present challenges to overcome, such as manufacturing hurdles and the possibility of unwanted toxicity.

### Micelles

The nanosized micelle system comprises amphiphilic molecules that can improve the solubility, bioavailability, and tumor targetability of hydrophobic anticancer drugs [64]. As spherical colloidal particles, micelles are self-assembled with a hydrophobic core to load poorly soluble compounds and a hydrophilic shell to achieve steric stability and less nonspecific uptake [65]. In recent years, pH-sensitive micelles have exhibited the ability to disrupt endosomal function for cytosolic drug delivery [66]. For example, a nanoscale poly(ethylene glycol)-b-poly(2-hexamethyleneimino ethyl methacrylate) (PC7A) micelle was developed as a pH-sensitive polymer with a seven-membered ring [67]. PC7A could induce innate immunity pathways through polyvalent interaction with another surface site of the STING protein, different from that of the STING-cGAMP binding pocket. Thus, PC7A NPs loaded with cGAMP synergistically generated robust pro-inflammatory chemokines [68]. Su et al. also synthesized a pH-responsive polymeric micelle to co-load with cGAMP and peptide antigens. After endocytosis, NPs were disassembled in the acidic endosome, facilitating the release of cGAMP into the cytosol for STING activation. Moreover, these NPs demonstrated high targeting specificity to APCs, effectively overcoming random dissemination of peptide antigens and reducing potential immune toxicity [69].

### Metal-based NPs

Metallic NPs are made in various forms, including pure metal, metal oxide, and metal salt [70]. Emerging evidence has revealed that metal NPs have the potential to serve as multipurpose agents. To deliver STING agonists, metallic ions, such as Fe^2+/3+^, Zn^2+^, and Mn^2+^, can assemble with CDNs into spherical NPs that act as vectors for targeted delivery. Among these metal ions, Mn^2+^ was identified to stimulate STING-independent immunity by inducing phosphorylation of TBK1 and the expression of NF-κB p65 in monocytes [71]. Sun et al. established a manganese-based nanoplatform by self-assembling CDA and Mn^2+^ through a coordination ligand. CDA-Mn-NPs showed superior cell uptake and cytosolic localization in bone marrow-derived dendritic cells (BMDCs), followed by increased IFN-β responses by more than 20 times, compared to a free admixture of CDA and Mn^2+^ [71]. Similarly, a STING agonist (MSA-2) was co-assembled with iron oxide-based copolymers, forming acid-ionizable iron NPs (INOPs). Under conditions similar to the acidic endosomal environment, INOP protonation was significant in initiating the dissociation and subsequent release of MSA-2. IONPs also led to intracellular reactive oxygen species (ROS) through the Fenton reaction, which amplified MSA-2-induced type I IFN production [72]. Moreover, Yang et al. engineered a nanoparticle (NP) loaded with CDA, featuring a hydrophilic core composed of zinc phosphate formed through coordination polymerization. Zn-CDA-NPs exhibited pharmacokinetic advantages over conventional liposome formulations. Specifically, Zn-CDA-NPs effectively attenuated CDA degradation in serum and prolonged CDA circulation half-life compared to a CDA-loaded liposome [73].

Furthermore, the remarkable properties of metal-organic framework (MOF) nanomaterials have received significant attention, leading to their exploration in diverse biological applications. Nanoscale MOFs are a class of porous materials composed of metallic ions or clusters interconnected by organic linkers [74]. The superior attributes of MOFs, such as specific surface area and porosity, high biocompatibility, and thermal stability, provide promising opportunities as carriers of the STING agonist [75–77]. Zhou et al. constructed a polymeric MOF (PMOF) NP that encapsulated a STING agonist (SR-717). For the regulated release of SR-717, a specific photosensitizer (meso-tetra(carboxyphenyl)porphyrin, TCPP) was incorporated into the PMOF to enable the controlled separation of SR-717 through singlet oxygen (^1^O_2_) mediation. Under light irradiation, TCPP generated ^1^O_2_, which broke thioketal bonds and further destroyed the PMOF structure to release SR-717. Consequently, ROS generation and the release of SR-717 from PMOF have demonstrated a synergistic effect, effectively enhancing the activation of the STING pathway [78].

### Mesoporous silica

As inorganic nanomaterials, mesoporous silica NPs (MSNPs) could be functionalized as an enhancement of endocytosis by targeted cells with less toxicity [79]. The electrostatic interaction between cationic NPs and the anionic phospholipid membrane of tumor cells has been shown to increase the penetration and distribution of NPs in TME [80]. To form a cationic MSNP, an amine-modified MSNP was designed, and it electrostatically binds to negatively charged CDG. The introduction of amine-modified MSNPs led to enhanced retention of CDG at tumor sites, in contrast to the rapid dispersion observed with free CDG [81]. Chen et al. also synthesized a CDG-loaded MSNP with a small diameter for its easy penetration through the tumor matrix. PEGylated modification and quaternary ammonium-modified cationic molecules were also introduced to these MSNPs, increasing the stability of blood circulation, tumor accumulation, and cellular uptake. Compared to free CDG, quaternary ammonium-based MSNPs loaded with CDG efficiently triggered STING activation in macrophages [82]. Similarly, Bielecki et al. also formulated protonatable primary and secondary amines on the surface of MSNPs, which facilitated the uptake of CDG by APCs and released those into the cytosol. Induction of pro-inflammatory IFN-β was also observed in these amine-functionalized MSNPs that wrap CDG in macrophages [83]. However, several drawbacks, such as small pore sizes for drug delivery, slow biodegradation, and long-term retention in normal tissue, restrict the application of conventional MSNPs. Therefore, Park et al., engineered a biodegradable MSNP characterized by a larger pore size (5–10 nm) and a thinner Si-O-Si matrix that allowed rapid degradation under physiological conditions. Further studies showed efficient cytosolic delivery of CDA incorporated in this wide-apertural MSNP, along with solid STING activation in monocyte-derived cells [84].

### Other nanocarriers

So far, polymeric nanomaterials have emerged as versatile tools to improve drug bioavailability or specific delivery at tumor sites [85]. Several frequently used polymers, such as poly(lactic-co-glycolic acid) (PLGA) and poly(beta-amino esters) (PBAEs), have been tested for the encapsulation of STING agonists [86–89]. Recently, some novel and reformative approaches have been performed to optimize the structure of polymeric NPs for encapsulating STING agonists. Semiconducting polymeric NPs (SPNs) have growing promise due to their high tumor penetration and specific drug release by external stimuli, such as sonodynamic conversion [90]. Jiang et al. exploited a sonodynamic SPN for loading MSA-2 and driving STING activation in head and neck squamous cell carcinoma. The semiconducting polymer skeleton could be an MSA-2 sonosensitizer through a ^1^O2 cleavable linker. Through sono-irradiation directed to tumor sites, MSA-2 could be accurately released in TME through cleavage of the diphenoxyethane bond. Unleashed MSA-2 further triggered IFN-β secretion through the phosphorylation of TBK1 and IRF3, thus increasing the activation of APCs. This in situ sono-driven STING activation action presents a novel strategy to provide immunomodulation with precise spatio-temporal control [91].

Recent studies have highlighted the increased penetration capacity of nanomaterials with morphologies of high aspect ratios [92]. Dane et al. thus employed a PEGylated lipid nanodisc (LND) with a flexible high-aspect ratio. The CDN prodrug was conjugated to LND and cleaved by cathepsin after cellular internalization into the endosome. Compared to the CDN-loaded liposome, CDN-loaded LND showed superior accumulation and penetration of CDNs into tumor sites and LNs in a mouse model of colorectal cancer [93]. However, using PEGylated NPs raises potential concerns about preexisting anti-PEG responses, such as allergic reactions, alerting developers to the side effects of exogenous modification on nanocarriers [94]. To improve the biosafety of drug carriers, Xu et al. introduced supramolecular interactions in the synthesis of nanomaterials. The hydrophilic CDG was assembled with hydrophobic 3′,5′-diOA-dC via supramolecular interactions, including hydrogen bonding and hydrophobic interaction, avoiding the use of a non-clinically exogenous carrier. Considering the biosafety, 3′,5′-diOA-dC can be metabolized to relatively safe ingredients, such as oleic acid and deoxycytidine, in vivo. Compared to free CDG, supramolecular CDG-NPs also showed increased accumulation and retention of CDG in murine melanoma tumor tissues, accompanied by increased secretion of type I IFNs [95]. Furthermore, extracellular vehicles (EVs) have been identified as natural NPs separated by lipid bilayers secreted from various cells, with favorable biocompatibility for drug delivery [96]. Jang et al. employed exogenously loaded EVs with CDNs. This led to substantial tumor retention and a notable increase in IFN-β production, exhibiting approximately 100 times higher potency than free CDNs [97].

## The promising immune responses caused by nanoencapsulation of single STING agonists

**Fig. 3:**
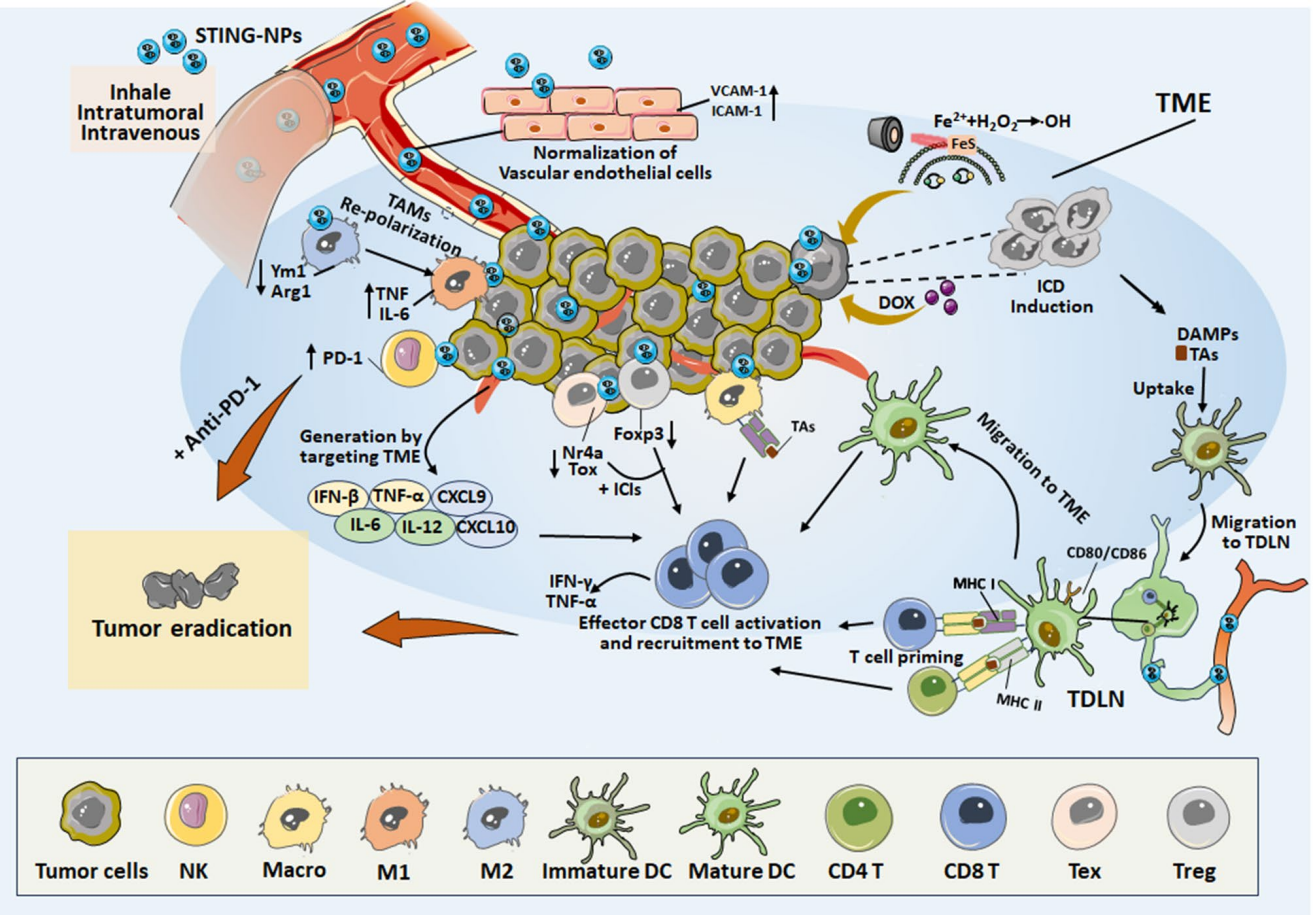
The modulation of STING-NPs on immune components of TME and LNs for anti-cancer immunotherapy

TME comprises host-derived microvasculature, stromal, and immune cells interacting with cancer cells. Some cancers are characterized by an immunologically “cold” nature, insufficiently infiltrating effector CD8^+^ T cells, and abundant immunosuppressive cell subtypes. The STING-associated pathway has recently emerged as a critical player in activating innate and adaptive immune responses. Innovatively, NPs integrated with STING agonists have been proposed as a novel approach to enhance antitumor effects. Unlike soluble forms, NPs loaded with STING agonists have demonstrated superior aspects, particularly in treating low-immunogenic cancers. In this summary, we discuss the modulation of immune components within the TME by encapsulating single STING agonists in NPs (Fig. 3).

### Secretion of cytokines

NPs carrying STING agonists show a solid potency to expand the release of pro-inflammatory cytokines and chemokines, which recruit CD8^+^ T cells infiltrating in TME, and strengthen immune responses in various cancers. In a murine model with glioma, ferritin-assembled NP loaded with SR717 improved IFN-β, TNF-α, CXCL-9, and CXCL-10 expressions in TME. In contrast to free SR717, mice exposed to SR717-ferritin-NP in the presence of glioma exhibited markedly increased levels of CD8^+^ T cell infiltrating, resulting in reduced tumor growth and extended survival [98]. In another colorectal mouse model, intravenous injection of Mn-CDA-NP significantly increased serum levels of IFN-β, TNF-α, CXCL-9, and CXCL-10, along with increased antigen-specific CD8^+^ T cell responses [71]. TDLNs are crucial sites for antitumor T cell priming and effective systemic immunity [99]. After treatment with polymeric cGAMP-loaded NPs, CXCL-10 expression in melanoma TDLN was upregulated, consistent with a significant increase in CD8^+^ T cell infiltration in TME [61]. Other cytokines, such as IL-6 and IL-12, were also increased by STING-NPs in TME, which promote the recruitment and activation of anticancer immune cells [54, 100]. Furthermore, STING-NPs improved the pro-inflammatory transpiration profiles of CD8^+^ T cells in TME. Intratumoral injection of the cGAMP-loaded polymersome significantly increased the frequency of TNF-α positive CD8^+^ T cells compared to using free cGAMP in melanoma TME [59]. The IFN-γ is secreted mainly by cytotoxic CD8^+^ T cells, which can mediate tumor rejection [101]. An et al. reported that CDG-loaded MSNPs improved the percentages of TNF-α positive CD8^+^ T cells and IFN-γ positive CD8^+^ T cells compared to those treated with free CDG, leading to the inhibition of tumor growth in a murine melanoma model [81]. These findings suggest that STING-NPs can induce cytotoxic CD8^+^ T cells capable of secreting pro-inflammatory cytokines, such as IFN-γ and TNF-α, potentially resulting in robust antitumor immune activities.

### DC activation and TA presentation

As essential components of APCs, DCs activate effector responses by presenting TAs to T cells [102]. In response to TAs, DCs undergo immunogenic maturation that confers up-regulation of molecules of the major histocompatibility complex (MHC) and co-stimulatory molecules (e.g., CD80 and CD86) [103]. Various NPs incorporating STING agonists have been well studied in the aspects of DC maturation and presentation of TAs. For example, in vitro research discovered that coculture of cGAMP-NP-treated neuroblastoma cells and BMDCs caused DC maturation with up-regulated expression of MHCII, CD86, and CD80 [60]. Liu et al. also reported an increased expression of CD86 and MHC-II in DCs within the TME of 4T1 (breast cancer) metastases-bearing lungs after inhaled treatment with cGAMP-loaded NPs [54]. To trigger systemic immunity, mature DCs can present exogenous antigens by cross-presenting MHC-I with CD8^+^ T cells [104]. Liu et al. further utilized the ovalbumin (OVA)-expressed melanoma murine model to investigate the cross-presentation of TA since the OVA peptide SIINFEKL can serve as an exogenous TA for tumor-specific T cell priming. After the combined therapy of irradiation (IR) treatment and cGAMP-loaded NPs, the TA complex (SIINFEKL-MHC-I molecules) was significantly improved in mature DCs that migrated to TDLNs, accompanying the expansion of tumor-specific CD8^+^ T cells [54]. Immunogenic cell death (ICD) is perceived as a type of cancer cell death that unleashes endogenous damage-associated molecular patterns (DAMPs), resulting in efficient tumor‐specific immunity [105]. Using an NIR light, a FeS_2_ and cGAMP photothermal-mediated liposome loading induced ICD by the generation of hydroxyl radical (·OH) at tumor sites. ICD-induced DAMPs increased calreticulin, HMGB1, and ATP, and accelerated DC maturation (CD80^+^CD86^+^). The combinational action of ICD and STING activation also increased CD4^+^ and CD8^+^ T cell trafficking to primary and distant tumors in a triple-negative breast cancer (TNBC) mouse model [56]. Doxorubicin (DOX) is a well-known chemotherapeutic agent to induce ICD [106]. Chen et al. developed a cGAMP-loaded liposome that captures TAs generated by DOX-induced ICD within the melanoma TME. These cGAMP-NPs additionally aided in transporting TAs to TDLNs for uptake by mature DCs. This led to robust activation of CD8^+^ T cells, resulting in significant suppression observed in both primary and rechallenged melanoma models [107].

### The reinvigoration of TAMs

TAMs are versatile immunocytes in the formation of TME and are often polarized into phenotypes M1 (classically activated) and M2 (alternatively activated) [108]. M1 phenotype macrophages tend to exert tumor-killing functions that trap, phagocytize and eliminate tumor cells, while M2 phenotype macrophages dampen T-cell activation and promote tumor progression [109]. NP-based STING agonists have been identified for intervention in the spectrum of repolarization of TAMs in vivo. In melanoma TME, cGAMP-loaded NPs reduced the differentiation tendency of M2 macrophages and downregulated an M2 marker CD206 [53, 59]. In another example, in situ treatment combined with DOX and NP-delivered cGAMP synergistically contributes to a shift of macrophages into M1-polarized subtypes in melanoma [107]. Further evidence indicates transcriptional modulation in M1/M2 phenotypes in which cGAMP-NPs improves the repolarization of M2 toward M1 macrophages through downregulation of M2-like genes (Ym1 and Arg1) and upregulation of M1-like genes (TNF and IL-6) in a murine TNBC model [100]. The other role of macrophages may be as APCs at the start of therapy. However, unlike DCs, they might not play a crucial role in generating sufficient antitumor efficacy. In vitro research showed that the coculturing of splenic isolated CD3^+^ T cells with TA-pulsed macrophages treated with cGAMP-NP B16F10 (melanoma) triggered remarkable secretion of IFN-γ, suggesting that NPs increased antigen presentation and T cell priming [100]. Yang et al. also reported that Zn-CDA-NPs were involved in antigen-specific CD8^+^ T cell responses depending on endogenous activation of TAMs by STING in colon cancers. The STING agonism of Zn-CDA-NPs promoted the functions of TAMs in the presentation of TAs by upregulating the expression of co-stimulatory factors (CD80 and CD86) and MHC-I. Meanwhile, Zn-CDA-NPs were found to effectively suppress antigen degradation by lysosomes through the downregulation of lysosomal enzyme-related genes in TAMs. This process plays a crucial role in ensuring the presentation of TAs to T cells [73].

### Targeting the vasculature

Tumor vasculature, characterized by an abnormal vascular structure, restricts immunotherapeutic agents that penetrate tumors and hinders the immune response against tumors [110]. Recently, targeting receptors in the tumor vasculature served as potential pharmacological targets for the design of STING-NPs. NPs with vascular ligands that target fibronectin, αvβ3 integrin, and P-selectin have been formulated to anchor STING agonists to APC-rich perivascular TME [111]. Furthermore, STING-NPs demonstrated enhanced accumulation and penetration within the TME by disrupting the tumor vasculature. This disruption transformed the TME into an immune-favorable environment [73]. In addition, structurally and functionally abnormal vasculature hinders antitumor immunity by restricting immune cells from migrating to TME. Tumor-associated endothelial cells (ECs) within the tumor vasculature have been recognized for their ability to downregulate the expression of cell adhesion molecules, potentially hindering the infiltration of T cells into tumors [112]. Bishop et al. revealed that intravenous treatment of cGAMP-loaded polymers upregulated cell adhesion molecules, such as VCAM-1 and ICAM-1, which normalized vascular ECs and promoted CD8^+^ T cell transmigration in renal cell carcinoma TME. Furthermore, normalization of ECs further triggered the infiltration of endogenous and adoptively transferred T cells in response to cGAMP-loaded NPs in orthotopic EO771 breast tumors [113].

### Other immune components of TME

Immunosuppressive cellular components in TME often limit efforts to restore a curative response [114]. Regulatory T cells (Tregs), a specialized subpopulation of T cells, suppress the activation and differentiation of helper CD4^+^ T cells and cytotoxic CD8^+^ T cells to inhibit anticancer immunity [115]. NPs loaded with STING agonists have been developed to block Treg recruitment in TME. For example, NPs containing cGAMP have been shown to down-regulate the expression of the Foxp3 transcription factor, facilitating a reduction in the Treg population within the neuroblastoma TME [54]. Similarly, a liposome with cGAMP delivery reversed IR-induced Treg infiltrations. It improved the CD8^+^ T/Treg ratio, suggesting effective prevention of NPs in Treg infiltration in TME [54]. Furthermore, exhausted T cells (Tex) are characterized by dysfunction of effector functions that contribute to failures in immunotherapy [116]. Yu et al. developed an NP composed of a neutral cytidinyl liposome and a cationic liposome (mix) incorporated with CDG for treatment of breast cancers. Compared to free CDG, intratumoral treatment of CDG/Mix-NPs significantly decreased Tex in TME. CDG/Mix-NPs also reversed the Tex by down-regulating the transcription factors Tox and Nr4a of Tex [117]. Furthermore, the action of immune checkpoints has been described to cause cytotoxic T-cell exhaustion and dysfunction [118]. Adaptive immune resistance could be generated, such as up-regulation of PD-L1 in tumor cells in response to STING activation [53]. To address these obstacles, STING-NPs were applied to synergize immune checkpoint blockade (e.g., anti-PD-1/anti-PD-L1, anti-CTL4) therapy, which reduced tumor growth, promoted long-term survival, and induced immunological memory [53, 59, 61].

NK cells, known as “tumor killers,” are generally involved in the innate immune response. Intratumoral activation of NK cells was observed within STING-NP treatment [84, 98]. Recent studies have revealed that PD-1/PD-L1 interactions inhibit NK cell responses [119]. Therefore, a combination of PD-1 antibodies and STING-NPs could activate NK cells to overcome resistance to immunotherapy. Nakamura et al. developed lipid CDG-loaded NPs that induced systemic activation of NK cells, and part of them obtained PD-1 expression. When combined with anti-PD-1 treatment, CDG-loaded NPs sensitized the antitumor effects of PD-1 blockade by promoting the capacity of NK cells to kill melanoma tumors [120]. Similar results revealed that PD-1 expression was elevated in NK cells during second-cycle treatment with lipid CDG-loaded NPs, further strengthening anti-PD-1 treatment against lung metastases in a mouse model with melanoma [121].

## Immunomodulation by co-delivery nanoplatform of STING agonists and antitumor agents

**Fig. 4:**
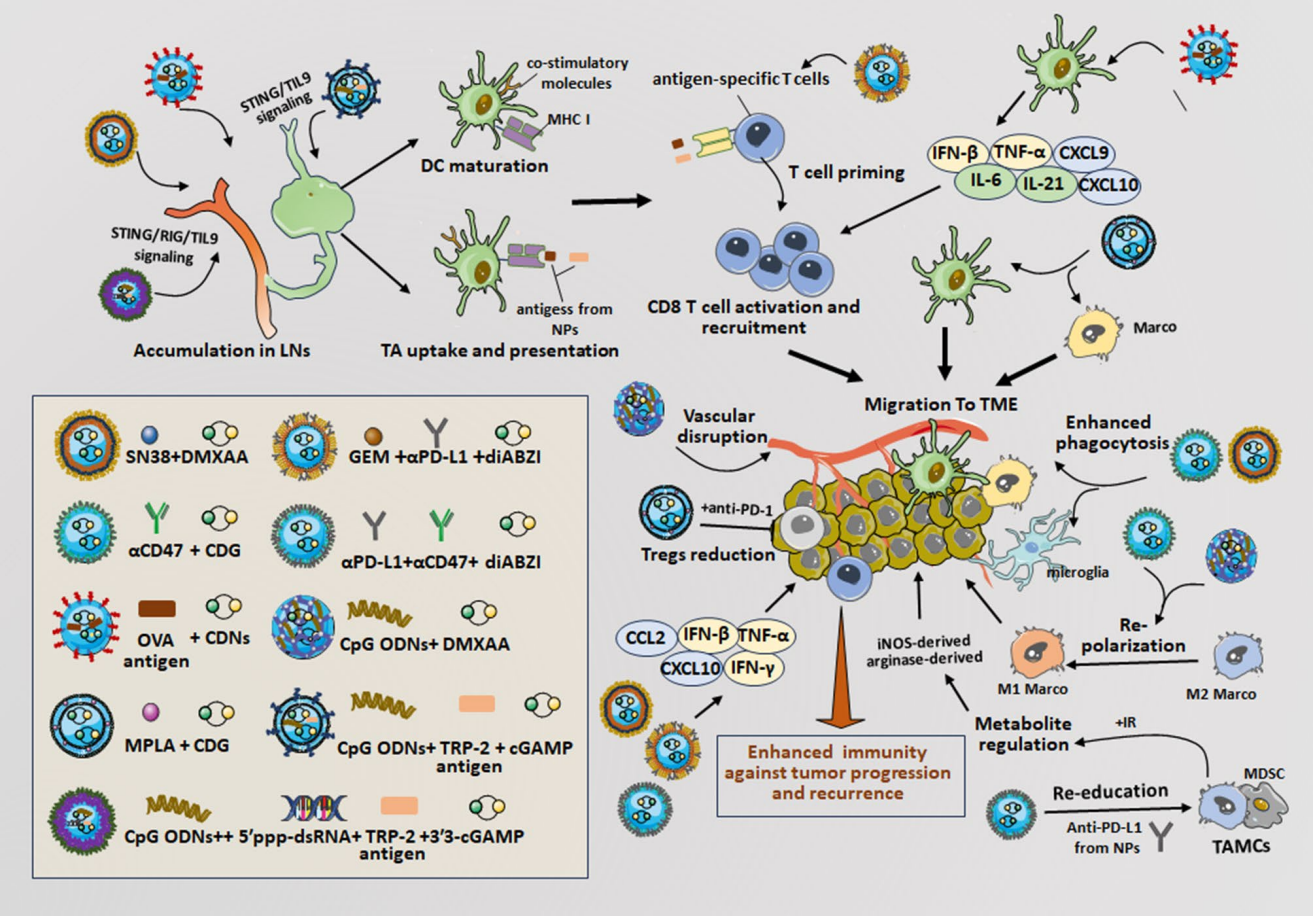
The anti-tumor immune responses induced by co-delivered NPs with STING agonists and anti-tumor agents. *Note*: CpG ODNs + cGAMP + TRP-2 particles are nanoporous (40–100 nm) microparticles for STING agonism

In recent years, the co-encapsulation of STING activators along with various immunotherapeutic agents into nanocarriers has received increased attention from researchers. Promising candidates for co-encapsulation include ICIs, peptide antigens, immune adjuvants, and chemotherapeutic agents. These combinations have the potential to synergize and enhance the antitumor effects of STING agonists. Therefore, we describe co-encapsulated nanoplatforms and explore their impacts on immune responses in cancer therapy (Fig. 4).

### Co-delivery of STING agonists and chemotherapeutic agents

Recent studies have shown that chemoimmunotherapy nanosystems provide an opportunity to reshape immunosuppressive TME through innate and adaptive immunities [118]. SN38 is an active metabolite of the topoisomerase I inhibitor irinotecan for treating colon or rectal cancers [122]. Zhao et al. screened SN38 as a potent candidate to stimulate activation of the STING pathway and production of type I IFNs in breast cancer cells. Furthermore, they constructed a polymeric NP for SN38 delivery that provoked innate immune responses, including induction of pro-inflammatory cytokines with activated DCs and NK cells. However, no significant changes in T cells were observed within the SN38-NP treatments compared to free SN38. It implies that a single encapsulation of SN38 in NPs may not be sufficient to provoke adaptive immune responses [123]. In another research by Liang et al., the addition of DMXAA encapsulation to SN38-loaded NPs (PEG-PSN38-PDEA) increased innate and adaptive immunity in a melanoma mouse model. Compared to NPs with a single loaded SN38, intravenous injection of DMXAA-SN38-NPs increased the secretion of pro-inflammatory cytokines, including IFN-β, IFN-γ, and TNF-α, in TME. Incorporation of DMXAA in SN38-NPs also amplified DC maturation and recruitment in TME and LNs, activating antigen-specific CD8^+^ T cells in B16-OVA murine models. Administration of DMXAA-SN38-NPs resulted in a notable increase in the IFN-γ positive effector CD8^+^ T cells and a higher ratio of CD8^+^/CD4^+^ infiltrating tumors. This combination led to a powerful suppression of melanoma growth [63].

### Co-delivery of STING agonists and ICIs

A growing body of evidence has recommended STING agonists as adjuvants to ICIs to synergize antitumor immunity [124]. Based on the concept of combined immunotherapy, co-delivery of ICIs and STING agonists via nanotechnology is prone to strengthen immune surveillance. Shi et al. designed a triple-combination immunogenic nanovesicle that included gemcitabine (GEM), αPD-L1, and diABZI (Gemcitabine-αPD-L1-STING agonist, GPS). The GEM prodrug, with functional moieties at the 4-(N) position, is covalently ligated to a biodegradable polymer through covalent linkage. αPD-L1 was then conjugated to polymer GEM nanovesicles that further encapsulated diABZI. The coating αPD-L1 on the GPS surface enhanced the interaction between cancer cells and increased the accumulation of intratumor NPs. It is worth noting that GPS maintains a preferable duration of TNBC and pancreatic cancer inhibition, and a significant reduction in metastatic challenges compared to joint treatment of these free drugs. In terms of its immunoenhancement properties, GPS treatment triggered DC maturation with elevated expression of CD80 and CD86, resulting in the highest secretion of IFN-γ and TNF‐α within the TME of TNBC. Furthermore, GPS also exhibited a robust antigen-specific T-cell response, coincident with potent memorized immunity against tumor rechallenges in the B16‐OVA melanoma model [125].

Immune checkpoints can also prompt cancer cells to readily evade phagocytosis of APCs. CD47 serves as a “do not eat me” signaling checkpoint, facilitating cancer cells to evade macrophage-mediated phagocytosis through its interaction with signal-regulatory protein alpha (SIRPα) [126]. A recent study has co-delivered a small interfering RNA targeting SIRPα (siSIRPα) and cGAMP in APCs by polymeric NPs. This study illustrates that the encapsulation of siSIRPα and cGAMP leads to a heightened expansion of CD8^+^ T cells infiltrating the tumor tissues. Consequently, this approach triggers comprehensive antitumor immune responses in melanoma mice [87]. These findings suggest that combining STING activation with inhibition of CD47-SIRPα signaling could synergistically improve APC phagocytosis within TME. Zhou et al. engineered a polymer layer encapsulating anti-CD47 antibodies (αCD47) and CDG by free radical polymerization. The surface modifications designed to mimic natural nicotinic acetylcholine substantially improved the blood-brain barrier (BBB) penetration and the glioma-targeting efficacy of CDG-αCD47-NPs. Compared to free αCD47, glioma growth was significantly inhibited by synergistically enhanced macrophages and microglial phagocytosis in the presence of CDG-αCD47-NPs. Moreover, CDG-αCD47-NPs re-educated TAMs and microglia to the M1 phenotype, thus increasing the infiltration of CD8^+^ cytotoxic T cells into glioma TME [127].

In another study, Peng et al. found highly expressed PD-L1 in tumor-associated myeloid cells (TAMCs) after IR pretreatment. TAMCs consist of TAMs and myeloid-derived suppressor cells, conferring immunosuppression and resistance to antitumor therapy [128]. To achieve TAMC-targeted therapeutic intervention, they designed bridge lipid NPs (B-LNP) containing diABZI and dual linkages of αCD47 and αPD-L1. The αCD47 in B-LNP inhibited CD47 overexpression in glioma cells, whereas the αPD-L1 was used to block up-regulated PD-L1 in TAMCs. The diABZI-loaded B-LNP was characterized by evident expression of pro-inflammatory genes related to interferon in TAMCs. Furthermore, the metabolic phenotypes of TAMCs were reprogrammed, manifesting as higher levels of iNOS-derived metabolites and reduced levels of arginase-derived metabolites. The reshaped TAMCs further affected the landscape of adaptive antitumor immunity in glioma TME and featured activation of CD8^+^ T cells along with increased levels of CXCL10 and CCL2. As a result, diABZI-loaded B-LNP favored RT-triggered antitumor immune responses, with over 60% tumor elimination in mice carrying glioma [129].

### Co-delivery of STING agonists and peptide antigens

DCs play a crucial role as coordinators in cancer therapy by facilitating antigen uptake and initiating antigen-specific T-cell priming responses [130]. Inspired by the endogenous mechanisms of the cGAS-STING pathway on DC activation, nanovaccines are formulated by co-assembling STING agonists and peptide antigens. The OVA-SIINFEKL peptides are common antigens that can be co-encapsulated with different STING agonists, such as CDG, 2’3’cGAMP, and DMXAA [131, 132]. Based on a pH-responsive modification of nanovaccines, cargos of STING agonists and OVA peptides escape the endosome and are released to the cytosol [69, 133]. Compared to free agonists or OVA peptides, nanovaccines with incorporated OVA and STING agonists were adequately accumulated in LNs for DC uptake and STING activation, and then DCs matured with higher expression of MHC and co-stimulatory molecules [69, 132]. The complex of MHC-I-SIINFEKL on the surface of DCs also increased markedly, suggesting the increased antigen presentation efficiency of co-delivered nanovaccines [132]. Additionally, co-delivered nanovaccines increase the generation of pro-inflammatory cytokines by DCs, such as IFN-β, TNF-α, CXCL-9, and CXCL-10, which promote the proliferation and recruitment of antigen-specific CD8^+^ T cells in melanoma TME [89]. Gao et al. also reported a cGAMP/OVA nanovaccine targeting Clec9a^+^ DCs with increased expressions of CXCL-10, IL-21, and IL-6 and primed responses of CD8^+^ T cells [134]. These studies have also shown that nanovaccine-induced CD8^+^ T cells further inhibit tumor growth in melanoma-bearing mice [89, 134]. Additionally, in a 4T1 breast tumor model, a nanovaccine containing DMXAA and OVA peptides suppressed lung metastasis in combination with αPD-L1 [132]. In another study by Su et al., cGAMP/OVA could generate durable antigen-specific T‐cell responses and inhibit rechallenged lymphoblastoma by producing CD8^+^ TEM cells and CD8^+^ TCM cells, especially abundant OVA‐specific CD8^+^ TEM cells [69]. These findings suggest that the rational design of nanovaccines with STING agonists and peptide antigens is expected to emerge for cancer immunotherapy.

### Co-delivery of STING agonists and TLR agonists

In addition to STING signaling, immune pathways such as Toll-like receptors (TLRs) and RIG-I‐like receptor ligands have been evaluated for their antitumor potencies [135]. TLRs, a family of evolutionarily conserved pathogen recognition molecules, are crucial regulators of innate immune responses. Among toll members, surface-located TLR4 can induce the activation of transcription factors NF-κB and IRFs to stimulate IFN-related inflammatory responses [136]. Considering the synergistic production of the IFN response through STING and TLR4 agonism, a lipid-bilayer NP was engineered for the co-delivery of CDG and a TLR4 agonist (monophosphoryl lipid A, MPLA). MPLA/CDG-NP lipid accumulated preferentially in the APC-rich perivascular regions of pancreatic tumors, with high-efficiency uptake by DCs. Compared to the combinational treatment of free agonists, MPLA/CDG-NPs significantly increased the proportions of DCs and macrophages in TME, indicating the tendency to activation in innate immunity [137]. Furthermore, the dual-agonist nanosystem (MPLA/CDG) combined with anti-PD-1 administration effectively curbed tumor growth in melanoma-bearing mice. This approach bolstered the presence of CD8^+^ T cells and significantly increased the CD8^+^ T cell to Treg ratio at the tumor sites. Furthermore, the synergistic treatment effectively targeted exhausted CD8^+^ T cells, resulting in heightened development of immunological memory in melanoma [138].

TLR9 is another member of the Toll family that is distributed in the endosome and serves as the primary sensor for the recognition and binding of CpG-DNA [139]. Cytosine-phosphate-guanine oligodeoxynucleotides (CpG ODNs) are TLR9 agonists that induce the production of pro-inflammatory cytokines for cancer therapy [140]. Chen et al. designed a multifunctional nanoplatform, DMXAA-CpG ODNs-MOF, with co-delivery of CpG ODNs and DMXAA via coordination bonds on MOF-801. Compared to CpG-MOF or DMXAA-MOF treatments, DMXAA-CpG ODNs-MOF treatment significantly improved immunosuppressive TME by reprogramming TAMs, inducing DC maturation, and disrupting the tumor blood vascular system in hepatocellular carcinoma [141]. Furthermore, extensive research has been conducted on utilizing multiple immune adjuvants and antigen delivery strategies [88, 142, 143]. Levy et al. established a polymeric nanosystem that was co-encapsulated with muti-immune adjuvants, including CpG ODN, 3′3-cGAMP, a RIG-I agonist (5′ppp-dsRNA), and a TRP-2 melanoma peptide. This multinano-structured platform containing immune agonist cocktails is highly internalized by DCs, and broadly enhances DC maturation in LNs and the spleen. This multinano platform facilitated the localization of TRP-2-specific T-cells in the TME of B16F10 lung metastasis, increasing the production of IFN-γ by effector CD8^+^ T cells [88]. Another approach involved co-encapsulation of cGAMP, CpG, and TRP-2 (cGAMP-CpG-TRP-2) within nanoporous (40–100 nm) microparticles, which could be more effectively distributed to the LNs than the smaller liposomes. The administered cGAMP-CpG-TRP-2 particles improved the maturation and migration of CD8^+^ and CD103^+^ DCs to LNs, leading to rejection in metastatic lung B16 tumors [143].

## Clinical trials

Current studies have revealed that STING agonists are novel candidates for antitumor immunotherapy in various phases of clinical trials. Until now, approximately 20 trials, including phase I and II, have been carried out to assess the clinical application of STING agonists [144]. For example, a phase I study evaluated the safety, pharmacokinetics, and efficacy of synthetic CDN (MIW815) in patients with advanced/metastatic cancers, providing evidence of systemic immune activation (NCT02675439) [145]. The efficacy and safety of intratumoral MIW815 combined with pembrolizumab are also being evaluated in adults with recurrent or metastatic head and neck squamous cell carcinoma positive for PD-L1 (NCT03937141). However, loading the delivery system with STING agonists is still scarce in clinical trials. A phase 1/2 open-label study (NCT04592484) is underway to investigate the dose increase, safety, and pharmacodynamic profile of exoSTING (CDK-002) in individuals with advanced/metastatic, recurrent, and injectable solid tumors. Furthermore, a microparticle with STING agonist delivery is being investigated in subjects with autoimmune encephalomyelitis and multiple sclerosis (NCT05705986).

## Perspective

Intralesional administration is a necessary route for free STING agonists, as the drug-like properties of CDNs are rapidly cleared from the circulation after intravenous administration [146]. Consequently, NP-based STING agonist carriers are designed to improve tumor accumulation and enhance STING agonism for systemic administration. So far, nanosystems with encapsulated STING agonists have demonstrated several advantages over free forms, including improved pharmacokinetic behavior and reduced side effects. For example, nanoencapsulation of cGAMP in a polymersome increased the half-life of cGAMP 40 times compared to the free form [61]. Studies have shown that NPs encapsulating STING agonists induce negligible systemic toxicity in preclinical models. Systemic treatment with STING-NPs did not produce apparent changes in body weight, blood indices, liver function, or histological damage to normal organs [56, 71, 93].

The synergistic approaches of STING agonists with ICIs, especially PD-1/PD-L1 blockade, could enhance the low immunogenicity of TME, thereby offering a promising combined strategy for cancer immunotherapy [147]. Nevertheless, systemic administration of free ICIs carries the risk of off-target problems, which can lead to severe adverse reactions [148]. To address this issue, co-encapsulated nanocapsules of STING agents and ICIs can release these agents at the tumor site, minimizing damage to normal tissues [125]. Similarly, other immune adjuvants, such as peptide antigens and CpG ODNs, suffer from poor pharmacokinetics due to their small size and electrostatic charges. Therefore, co-delivery nanosystems are ideal for co-assembling CDNs and other immune adjuvants in immunomodulatory tissues and cells, where NPs can exert antitumor immunity [69]. All of the evidence above suggests that STING-NPs hold promise for effective cancer immunotherapy with minimal nonspecific immunotoxicity. However, more research is needed to determine the optimal dose of STING-NPs, investigate the extent of the systemic distribution, and monitor for potential toxicities.

In addition to nanoplatforms encapsulated with regular STING agonists, other nanoagonists can also induce STING activation through various approaches. One approach involves utilizing dsDNA to create nanoparticles, capitalizing on the inherent sensing capabilities of the cGAS-STING pathway. In a 4T1 mouse model, treatment with dsDNA-loaded nanoparticles induced IFN-β production and increased infiltration of effector T cells [149]. Furthermore, specific chemotherapeutic agents can indirectly activate STING-related pathways by causing DNA damage in cancer cells. For example, Cao et al. documented a nanoparticle co-assembled with the DNA-targeted drugs cisplatin and camptothecin. Responding to ROS, cisplatin and camptothecin were released at the tumor site, triggering activation of the cGAS-STING pathway through double DNA damage. This led to the maturation of DC cells and the recruitment of CD8^+^ T cells in a mouse model of colorectal cancer [150]. Another telomere-targeting drug, 6-thio-2’-deoxyguanosine (6-thio-dG), can also induce DNA damage in tumor cells. Qin et al. developed a photodynamic nanodrug that simultaneously delivered chlorin e6 to induce immunogenic cell death, celecoxib to attract DCs, and 6-thio-dG for STING pathway activation. Each component of the nanoplatform played a unique role, resulting in a robust DC-initiated immune response against colorectal tumors [151].

Recent research has discovered that metal ions, particularly Mn^2+^, can enhance the sensitivity of cGAS to dsDNA and increase the binding affinity between cGAMP and STING [152]. Several studies have shown that Mn^2+^-based nanoparticles can activate immune responses and strengthen antitumor immunotherapy. For example, Zheng et al. developed a Prussian blue (PB)-mediated photothermal nanodrug formulation with MnOx and Mn^2+^-doped PB (MnPB-MnOx). The enriched Mn^2+^ promoted cytokine secretion through cGAS-STING activation, leading to DC maturation and transport to TDLNs for adaptive immune responses in a 4T1 mouse model. MnPB-MnOx also facilitated the release of TAs from tumor cells through the generation of ROS catalyzed with MnOx under NIR radiation, further promoting antigen-specific immune responses [153]. In another example, nanoparticles containing MnO_2_ and αPDL1 encapsulation improved DC maturation and cytotoxic CD8^+^ T cell infiltration by amplifying the STING signal, resulting in profound antitumor effects against metastasis in CT26 tumor models [154]. Additionally, Xu et al. presented a nanoplatform co-loading CDA and Mn2+, which exhibited versatile capabilities in addressing both cancer and SARS-CoV-2 infections through the restoration of immune responses [155].

The primary objective of exploring and developing nanodrugs for cancer is to translate them into clinical applications. However, the path of STING-NPs toward clinical translation still faces several hurdles at various stages. Firstly, the potential toxicity and side effects might be raised from the nanocarriers of STING-NPs, such as cationic materials or metal-based architectures. The interactions between these nanocarriers and physiological tissues may cause unwanted outcomes, such as formation of thrombi, cytotoxicity, and genotoxicity [95, 156, 157]. Secondly, NPs stability can be affected by biomolecules and components suspended in circulation. Upon contacting with these compositions in blood, NPs could be recognized by innate immune system to accelerate their clearance [158]. While modifications on STING-NPs are employed to extend their circulation and improve delivery, there is a challenge in balancing the stable loading, targeted location and biological responses [159]. Thirdly, although some STING-NPs have demonstrated favorable stability, distribution and efficacy in animal models, some of them may fail to produce attractive effects in human bodies [160]. The gaps between animal and human studies are nonnegligible, leading to distinct behavior and functionality of nanomedicines. Thus, further directions should be attached importance to understand and overcome these obstacles in cancer management. To achieve safe and reliable nanoplatforms, the optimal of size, dosage and surface charges of NPs are crucial for future researches. Moreover, it is notable to streamline the NP formulation complexity and ensure reproducibility, so that NPs can be easily characterizable for evaluation in clinical trials. A comprehensive exploration of the pharmacokinetics, retention, and efficacy of the formulated STING-NPs is also essential for their eventual clinical translation. Overall, it is foreseeable that through meticulous design and safety assessment, STING-NPs hold potential as a promising strategy in cancer immunotherapy.

## Data Availability

Not applicable.

## Abbreviations

ICIs: immune checkpoint inhibitors
TME: tumor microenvironment
APCs: antigen-presenting cells
DC: dendritic cell
NK cells: natural kille cells
IFN: interferon
TNF-α: tumor necrosis factor-alpha
cGAS-STING: cyclic GMP-AMP synthase -stimulator of interferon genes
cGAMP: cyclic GMP-AMP
TBK1: TANK binding kinase 1
IRF3: interferon regulatory factor 3
NF-κB: nuclear factor-κB
Nanoparticles: NPs
STING-NPs: STING agonist-encapsulated NPs
CDNs: cyclic dinucleotide
CDG: cyclic dimeric guanosine monophosphate
CDA: cyclic dimeric adenosine monophosphate
NIR: near-infrared
PEG-DBP: poly(ethylene glycol)-block-[(2-diethylaminoethyl methacrylate)-co-(butyl methacrylate)-co-(pyridyl disulfide ethyl methacrylate)
INOPs: ionizable iron NPs
ROS: reactive oxygen species
MOF: metal-organic framework
MHC: major histocompatibility complex
TAs: tumor antigens
IR: irradiation
ICD: Immunogenic cell death
DAMPs: damage-associated molecular patterns
DOX: doxorubicin
TAMs: Tumor-associated macrophages
Tregs: regulatory T cells
Tex: exhausted T cell
TLRs: Toll-like receptors
